# 8.2 ka event North Sea hydrography determined by bivalve shell stable isotope geochemistry

**DOI:** 10.1038/s41598-019-43219-1

**Published:** 2019-05-01

**Authors:** Juan Estrella-Martínez, Philippa L. Ascough, Bernd R. Schöne, James D. Scourse, Paul G. Butler

**Affiliations:** 1 School of Ocean Sciences, Bangor University, Askew St., Menai Bridge, LL59 5AB UK; 2 NERC Radiocarbon Facility, Scottish Universities Environmental Research Centre, Rankine Avenue, Scottish Enterprise Technology Park, East Kilbride, G75 0QF UK; 3 Institute of Geosciences, University of Mainz, J.-J.-Becher-Weg 21, D-55128 Mainz, Germany; 4 College of Life and Environmental Sciences, University of Exeter, Penryn Campus, Penryn, TR10 9FE UK

**Keywords:** Palaeoceanography, Palaeoclimate

## Abstract

The abrupt 8.2 ka cold event has been widely described from Greenland and North Atlantic records. However, its expression in shelf seas is poorly documented, and the temporal resolution of most marine records is inadequate to precisely determine the chronology of major events. A robust hydrographical reconstruction can provide an insight on climatic reaction times to perturbations to the Atlantic Meridional Overturning Circulation. Here we present an annually-resolved temperature and water column stratification reconstruction based on stable isotope geochemistry of *Arctica islandica* shells from the Fladen Ground (northern North Sea) temporally coherent with Greenland ice core records. Our age model is based on a growth increment chronology obtained from four radiometrically-dated shells covering the 8290–8100 cal BP interval. Our results indicate that a sudden sea level rise (SSLR) event-driven column stratification occurred between ages 8320–8220 cal BP. Thirty years later, cold conditions inhibited water column stratification but an eventual incursion of sub-Arctic waters into the North Sea re-established density-driven stratification. The water temperatures reached their minimum of ~3.7 °C 55 years after the SSLR. Intermittently-mixed conditions were later established when the sub-Arctic waters receded.

## Introduction

**Figure 1:**
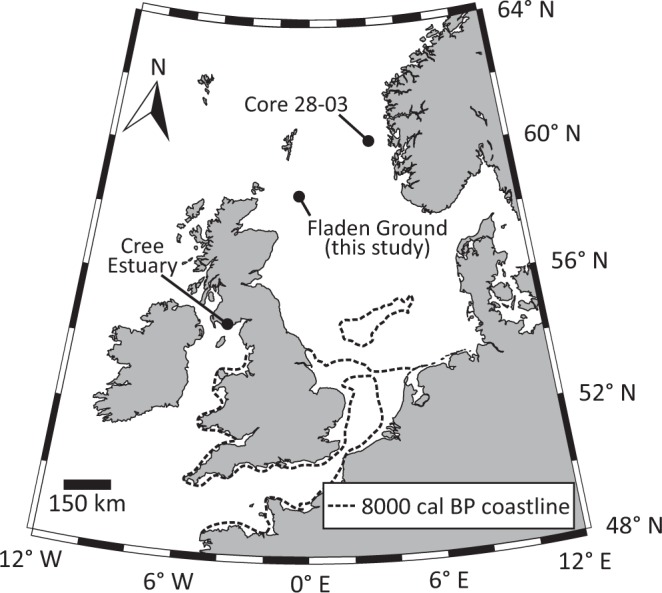
Approximate location of the sites mentioned in this work. The dashed line represents the approximate location of the coastline in 8000 cal BP^49^.

The 8.2 ka (before 1950 CE) cold event is usually defined by lower stable oxygen isotope values and a reduced ice accumulation rate in Greenland ice cores^1^. Although this event has been extensively described in the context of the Greenland ice cores and across the wider North Atlantic^1–5^, its expression in the Atlantic shelf seas is less well documented^6,7^. Here we present an annually-resolved reconstruction of the environmental conditions prevalent in the northern North Sea (Fig. 1) based on stable isotope geochemistry from radiocarbon-dated ocean quahog (*Arctica islandica*) shells centred around the 8.2 ka event.

The shell of the bivalve mollusc *A. islandica* is a key annually-resolved marine climatological archive for the North Atlantic margins^8^. The shells of *A. islandica* are common in the fossil record and readily dated by radiocarbon. The stable oxygen isotope composition (δ^18^O) of the shell reflects the δ^18^O of the water where the animal lived^9^ while the stable carbon isotope composition (δ^13^C) of the shell shows a consistent offset from the δ^13^C of the dissolved inorganic carbon of the water column^10,11^. Robust growth increment chronologies can be built from the annual increments in the *A. islandica* shell^12–15^, providing an accurate and annually-resolved chronological template for stable isotope data.

With this study we aim to determine the timing and causes of water column stratification in the North Sea in and around the 8.2 ka event, to reconstruct water temperatures during the 8.2 ka event and to establish an order of events registered in the North Sea in and around the 8.2 ka event. By comparing our reconstruction against Greenland ice core records we will also provide evidence against the hypothesis of a large asynchrony between the International Radiocarbon Calibration Curve (IntCal13) and the Greenland Ice Core Chronology 2005 (GICC05)^16^. As the first attempt to apply high resolution molluscan sclerochronological techniques to determine early Holocene environmental conditions this contribution constitutes a novel application of *A. islandica* sclerochronology.

## Radiocarbon Calibration and Chronologies

**Figure 2:**
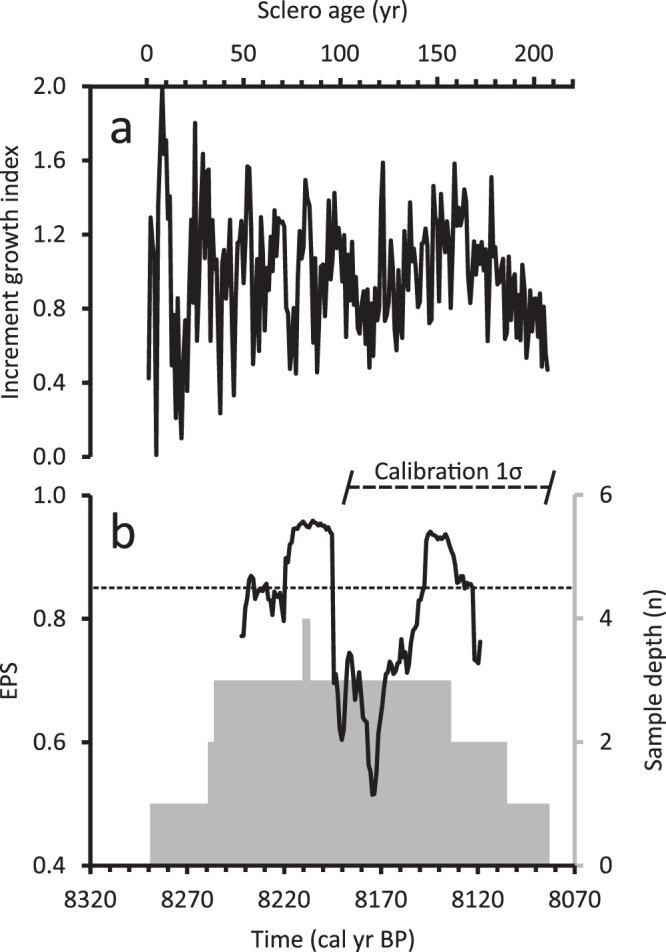
8.2kC and associated statistics. Growth increment chronology produced from the shells collected at the Fladen Ground (**a**). The chronology has a total sample depth of four shells and an average EPS of 0.81 (**b**).

The 2σ calibrated radiocarbon ages of our samples place the age of death of the bivalves into two temporal ranges: 8960–8540 cal BP and 8350–7990 cal BP (Table 1). For ease of analysis, we arbitrarily selected the median calibrated age for each shell as a reference point upon which to base the results and discussion. The reader is cautioned, however, that the calibration ranges are not necessarily normally distributed. Based on this assumption, our chronologies cover the intervals of 8860–8690 cal BP with possible range of ±164 years (8.7 ka chronology) and 8290–8080 cal BP (sclero ages 1–207) with possible range of ±106 years (8.2 ka chronology, “8.2kC”, Fig. 2a). The ±106 yr age range is applicable to all further references to dates derived from 8.2kC. The temporal uncertainty of the calibrated ranges rules out the possibility of merging the two floating chronologies into a single one. We thus made the choice of concentrating this investigation on 8.2kC. The 8.7 ka chronology and statistics can be found in the Supplementary Information.

**Table 1:** Radiocarbon dating results and calibration.

Laboratory ID	Shell ID (010…)	Radiocarbon age ± 1σ (^14^C yr BP)	Time gap (yr)	2σ calibrated range (cal yr BP)	Bayesian range (cal yr BP)
SUERC-8459	705	8356 ± 28	9	8990–8650	8960–8640
SUERC-8314	711	8332 ± 28	15	8970–8630	8950–8630
SUERC-8056	653	8306 ± 29	58	8950–8600	8940–8610
SUERC-8060	655	8275 ± 28	6	8930–8570	8880–8550
SUERC-8277	671	8247 ± 27	9	8880–8540	8880–8550
SUERC-8272	669	8231 ± 29	—	8860–8510	8870–8540
SUERC-8290	682	7794 ± 24	78	8320–8060	8350–8140
SUERC-8063	658	7810 ± 25	24	8340–8100	8270–8060
SUERC-8065	660	7801 ± 29	22	8330–8070	8250–8040
SUERC-8292	684	7752 ± 23	—	8280–8020	8200–7990

The average expressed population signal (EPS)^17^ score of 8.2kC is 0.81 and the 30-yr running EPS lies above the 0.85 threshold in sclero ages 52–61, 71–95, and 143–168 for a total of 49% of the calculated window (Fig. 2b). The lowest EPS score occurred when multiple specimens settled within a short time span combined with times of extended lower than average increment growth in other shells already contained in the chronology.

## Annual Stable Isotope Geochemistry

**Figure 3:**
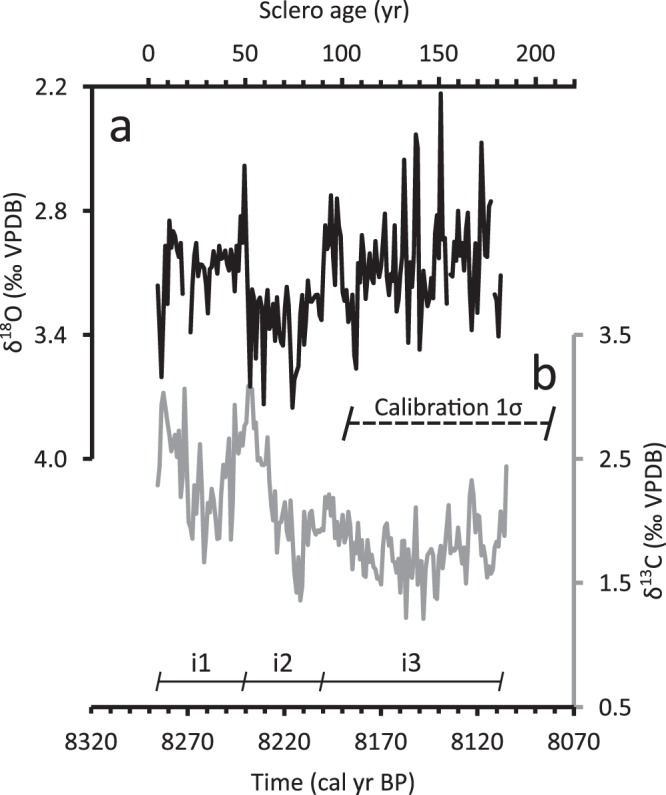
Stable isotope geochemistry. Annual weight-averaged δ^18^O (**a**) and δ^13^C (**b**) results. Notice inverted axis in **a**. Values in a can be divided into three intervals that show distinct average values and variance: Sclero ages 5–50 (i1), 51–90 (i2), and 91–186 (i3).

We obtained annual δ^18^O and δ^13^C results from three shells in 8.2kC covering sclero ages 5–186, equivalent to the interval 8286–8105 cal BP (Fig. 3a,b). We achieved a temporal overlap of 13 years between the first and second shell and an overlap of 21 years between the second and third shell. We were not able to extend the isotopic series to cover the entirety of the chronology as single-increment sampling becomes increasingly difficult in later ontogeny. Following our sample-rejection convention, we obtained 175 out 182 years sampled for δ^18^O but complete temporal coverage for δ^13^C. The composite (non-averaged) δ^18^O results fall within the range of 3.87‰ to 2.23‰, the former occurring in sclero age 107 and the latter in sclero age 152. Similarly, the composite δ^13^C results fall within the range of 3.08‰ to 1.15‰, the former occurring in sclero age 52 and the latter in sclero age 106.

The weight-averaged δ^18^O results (Fig. 3a) can be divided into three intervals that show distinct average values and variance: sclero ages 5–50 (i1), 51–90 (i2), and 91–186 (i3). i1 has an average of 3.07‰, a variance of 0.03‰^2^ and shows a small but significant positive trend of 0.04‰ decade^−1^. i2 shows an average of 3.34‰ and a variance of 0.03‰^2^. Finally, i3 has an average δ^18^O value of 3.04‰ and a variance of 0.05‰^2^. The δ^18^O values in all three intervals are normally distributed after the removal of extreme values: the maximum value in i1 (W = 0.94, p = 0.05), the three maxima in i2 (W = 0.94, p = 0.05) and the minimum in i3 (W = 0.98, p = 0.09). A two-tailed t-test on the modified values shows that the i1-i2 and i2-i3 changes in average are significant but the changes in variance are not (F-test).

Using the same interval definitions, the weight-averaged δ^13^C results (Fig. 3b) also show distinct average values and variance. i1 has an average of 2.39‰ and variance of 0.13‰^2^. i2 shows an average of 2.17‰, a variance of 0.21‰^2^ and a significant linear trend of −0.03‰ yr^−1^. Finally, i3 has an average δ^13^C value of 1.79‰, a variance of 0.05‰^2^ and no significant linear trend. The δ^13^C values in all three intervals are normally distributed without alteration and a two-tailed t-test shows that the i1-i2 and i2-i3 changes in average are significant but only the i2-i3 change in variance is significant (F-test).

**Figure 4:**
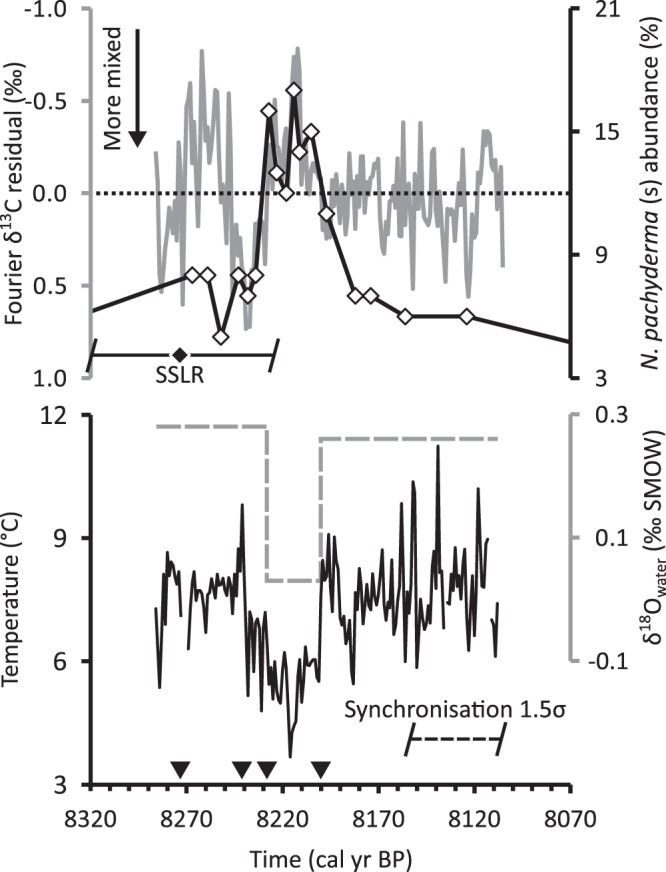
Fourier δ^13^C residuals (solid grey line, *inverted axis*) compared with the *N. pachyderma* (s) abundance record from core 28–03^6^ (white diamonds connected by black line), δ^18^O_water_ and temperature reconstruction. The residuals can be interpreted as a relative measure of water column stratification and are shown to be responsive to sudden sea level rise^18^ (black diamond and whiskers) and changes in δ^18^O_water_ (dashed grey line). Assumptions from the *N. pachyderma* (s) results can be used to reconstruct water temperature from the data generated in this study (solid black line). The black triangles depict the time when major climatic events were registered at the Fladen Ground (see text).

The Fourier δ^13^C residuals (Fig. 4) show distinct average properties in the defined intervals as well. i1 has an average of −0.05‰ and variance of 0.12‰^2^. i2 shows an average of −0.06‰ and a variance of 0.14‰^2^. Finally, the residuals show an average value of 0.04‰ and a variance of 0.04‰^2^ in i3. The residuals are normally distributed in all three intervals without alteration. The two-tailed t-test shows no significant in change in average and an F-test shows only the i2-i3 change in variance to be significant.

The calibrated age of interval i1 overlaps with a SSLR event identified at the Cree Estuary^18^ (black diamond and whiskers in Fig. 4). The possible age range for the SSLR event also overlaps with the calibrated age of i2 which coincides with a sudden *Neogloboquadrina pachyderma* (sinistral coiled, “s”) abundance increase in the far-north North Sea which is identified as the 8.2 ka event^6^ (average re-calibrated 2σ temporal range: ±158 years, Fig. 4). During i2, the maximum (minimum) δ^18^O (Fourier δ^13^C residuals) correspond with the maximum *N. pachyderma* (s) abundance in the core 28–03^6^. By the start of i3 the *N. pachyderma* (s) levels in the North Sea had already returned to their background levels.

## Discussion

The EPS in the newly developed chronologies does not generally achieve the recommended threshold^17^ of 0.85. The low EPS values shown in 8.2kC are likely due to the erratic juvenile growth of two specimens that make up its mid-section combined with lower than average shell growth in the other specimens. One way to avoid this problem is to disregard the first 20–30 years of growth when constructing *A. islandica* chronologies^14^ but the exclusion of juvenile years in this study would have meant a shorter chronology and a rather extended initial interval where the chronology would have been represented by only one shell.

Another possible explanation of the lower EPS values is growth increment counting errors where the sample depth is low. It has been shown that a 1% counting error rate can induce high EPS variability while a 5% error rate induces a continuous decrease in EPS for the entirety of the series^19^. Given the relatively high average EPS values for 8.2kC and the fact we observe EPS recoveries through our record, we estimate our counting error rate to be between 1 and 5%. This allows us to confidently base our stable isotopic results on the age model provided by the growth increment chronology.

When interpreting *A. islandica* stable isotope data we must consider the possibility that the organism is actively controlling the isotope composition of its shell (“vital effect”). There is unambiguous evidence that this is not the case for δ^18^O^9,20^. The evidence is less clear in the case of δ^13^C. Two studies arrived at conflicting results when examining the *A. islandica* vital effects on δ^13^C^20,21^. However, an analysis of 21 shells from Iceland found that vital effects represent only a small component of the δ^13^C variability in the first 30 years of the animal’s life^22^. We can therefore make palaeoenvironmental interpretations using both δ^18^O and δ^13^C data.

The greater temporal resolution provided by 8.2kC allows the close examination of the environmental conditions in and around the 8.2 ka event in the North Sea. During i1 we observe relatively stable δ^18^O values that become slightly higher near sclero age 20, equivalent to 8270 cal BP (Fig. 3a). The change is easier to observe in δ^13^C and in the Fourier δ^13^C residuals (Fig. 4) which show relatively large ^13^C depletions during the same sclero ages. The slight positive δ^18^O deviation can be interpreted either as a modest cooling, a change in δ^18^O_water_ or both. This is difficult to assess since there are no quantitative water-mass-independent temperature reconstructions for the North Sea with comparable temporal resolution for the temporal interval in question.

The changes in stable carbon isotope composition starting in i1, however, can be interpreted as the onset of stratification in the northern North Sea. This is because the *A. islandica* data represents a benthic record. During stratified conditions, limited vertical mixing results in a poorly ventilated deeper layer. Because primary production is limited to the shallow photic zone, the deeper layer is enriched in ^12^C. Shell carbonate precipitated under such conditions would, hence, have a lower δ^13^C value^23–25^. Large and consistent deviations in the Fourier δ^13^C residuals emphasise the higher frequency (sudden) changes in water stratification that can otherwise be masked by the gradual increase in sea level during the early Holocene.

The sudden North Sea water stratification was likely caused by the SSLR identified at the Cree Estuary. This SSLR event, attributed to the second drainage of the Lake Agassiz-Ojibway, had an estimated 0.4 m magnitude at the Cree Estuary^18^. The SSLR date range (8270 ± 53 cal BP, 3σ, Fig. 3) overlaps with the calibrated date when the Fourier δ^13^C residuals suggest stratified conditions initiated at the Fladen Ground during i1 (8270 cal BP, Fig. 3c). Given modern flushing times, northward Irish/Hebridean Sea currents^26,27^ and the trajectory of the meltwater pulse suggested in ref.^18^, it is highly likely that these two events are coeval or that they occurred within a year of each other. Synchronising these two events on the same age has the effect of reducing the 8.2kC dating range to ±53 years.

The sudden stratification event of our SSLR-synchronised Fourier δ^13^C residuals during i2 can be put in context when we compare our record to the North Sea age-recalibrated *N. pachyderma* (s) series^6^. The authors of that study determined that a brief incursion of sub-Arctic waters pushed the 20% abundance *N. pachyderma* (s) isoline towards the North Sea, extending to the Norwegian west coast and estimated a minimum water temperature decrease of 2 °C. Background conditions broadly similar to modern day were established upon the retreat of the sub-Arctic waters^6^. We can use this water advection/retreat and temperature drop assumptions to partially disentangle the temperature and water mass components of the δ^18^O proxy in our *A. islandica* shells. Using an updated version of the classical palaeotemperature equation^28^ (Supplementary Information) with the modern North Sea average^29^ δ^18^O_water_ value (0.26‰ SMOW) we can determine the i3 water temperature. We can then use the estimated 2 °C lower temperatures during the incursion of sub-Arctic waters to determine the average δ^18^O_water_ during that interval and repeat the procedure to determine the temperature and δ^18^O_water_ prior to the sub-Arctic water incursion (Fig. 4). Our estimated average δ^18^O_water_ (0.03‰ SMOW) during the sub-Arctic water incursion into the North Sea suggests that the 20% abundance *N. pachyderma* (s) isoline laid on fresher waters than on present day. This is in agreement with fresher waters being prevalent in higher latitudes during the 8.2 ka event^30^ which, in turn, point to a weakened Atlantic Meridional Overturning Circulation^2,31^.

By synchronising the first sudden stratification event observed in our Fourier δ^13^C residuals with the Cree estuary SSLR we can infer the order of events that caused the temperature and δ^18^O_water_ changes (events indicated by black triangles in Fig. 4) in the North Sea: In 8270 ± 53 cal BP a SSLR caused stratified conditions in the northern North Sea. This SSLR was caused by the second drainage of Lake Agassiz-Ojibway^32–35^ which forced the 8.2 ka event 30 yr later. This cold event inhibited summer stratification at the Fladen Ground and is associated with the southward extension of sub-Arctic waters. An incursion of fresher sub-Arctic waters into the North Sea (~0.03‰ SMOW) occurred 44 years after the SSLR, bringing with it the 20% *N. pachyderma* (s) isoline and a return to water-density driven stratified conditions. The water temperatures in the northern North Sea reached their minimum of 3.7 °C 55 years after the SSLR. Shortly thereafter, the sub-Arctic waters receded and background conditions with intermittently mixed warmer waters were established 70 years after the SSLR.

Due to the lack of highly-resolved marine archives during the 8.2 ka event, we are prevented from carrying out a direct comparison with adjacent oceanic records. We can, however, compare our reconstruction to the semi-annual Greenland ice core records^4^ and to a sub-annually-resolved speleothem δ^18^O record from central/east China^36^ that shows dry conditions during the event. These two records co-vary to a high degree and their age uncertainties are comparable with our reconstruction. The order of events and temperature variability suggested by our record is consistent with the “central 8.2 ka event” observed in Greenland ice cores^4^, both showing a duration of 70 years. The coldest conditions in both records are offset by 27 years in their respective age models, well within our possible radiocarbon dating range of ±106 years and the SSLR-synchronised dating range of ±53 years. If the offset is manually eliminated then the two records show a significant positive correlation (r = 0.39, p < 0.01). On the other hand, the driest conditions suggested by the U/Th-dated Chinese speleothem record and the coldest temperatures in our reconstruction are offset by 40 years, still within the SSLR-synchronised dating range. The dry event recorded in China also has a duration of 70 years. If the offset is manually eliminated then our temperature reconstruction shows a significant correlation with the Chinese δ^18^O record of −0.55 (p < 0.001).

The high level of synchrony between our temperature reconstruction and the Greenland ice core record implies that the date proposed in ref.^4^ as the start of the 8.2 ka event would differ from the SSLR-driven stratification in the North Sea by 1 year. Similarly, the start of the Greenland “central event” and the incursion of sub-Arctic waters into the North Sea would differ by 4 years. The small offset between our reconstruction, the Greenland record and the Chinese speleothem record and the high level of similarity between the three suggests that the GICC05 and the IntCal13 calibration curves are more synchronous than other authors have suggested^16^.

In this study we have developed two *A. islandica* c.200-year-long floating GICs. Radiocarbon dating places these chronologies in the early Holocene and we developed annually-resolved δ^18^O and δ^13^C series from one of them which is centred around the 8.2 ka event. These series are currently the only high or mid-latitude marine records with such a high temporal resolution and they suggest that two major water column stratification episodes, separated by 44 years, occurred in the North Sea between 8320 and 8220 cal BP. The first episode is likely to be coeval with a sudden sea level rise registered at the Cree Estuary caused by the second drainage of Lake Agassiz-Ojibway while the second episode was caused by the incursion of sub-Arctic waters into the North Sea. By utilising existing assumptions about water temperature and water mass changes we were able to determine that the bottom water temperature was ~3.7 °C between 8270 and 8160 cal BP, during the height of the 8.2 ka event. Finally, we determined that the drainage of Lake Agassiz-Ojibway, the initial temperature drop, the incursion and the recession of sub-Arctic waters into the North Sea were separated by 30, 14 and 26 years, respectively.

Our reconstruction is highly coherent with Greenland ice core records and provides an insight into the expression of the 8.2 ka event in shelf seas. By using the stratigraphic template provided by the *A. islandica* GIC, it is possible to reconstruct the chronological order of major events with high precision. This information is significant in understanding the reaction times to perturbations of the Atlantic Meridional Overturning Circulation.

## Methods

The *A. islandica* shells used in this study were collected from the Fladen Ground in the northern North Sea at 58.831° N, −0.356° E at a depth of 115 m. The collection was acquired by the RV *Scotia* as part of the EU HOLSMEER^13^ project in 2001. A total of ten shells with heights >70.0 mm and taphonomic characteristics that suggested the specimens were ancient^37^ were selected. These were sectioned using standard sclerochronological procedures^13,14^. Polished shell sections were viewed under reflected light, and the imaging software package ImagePro Premier 9.1 was used to identify and measure the growth increments in the outer layer of the ventral margin of the shell.

Radiocarbon dating was carried out on the edge of the ventral shell portion (deposited in late ontogeny) of the ten shells. To achieve the optimal carbonate mass required for accelerator mass spectrometry dating, it was necessary to cut samples that integrate the final years of growth. Precisely determining the number of years integrated in each sample was not possible since the age and length are related by a non-linear function^38^. We used a working approximation of 30 years. The material was submitted for preparation and measurement to the Natural Environment Research Council Radiocarbon Laboratory at East Kilbride, United Kingdom, where it was processed using the methods described in ref.^14^ prior to ^14^C analysis.

Calibration of radiocarbon ages was achieved with OxCal 4.3^39^. We used the Marine13 calibration curve^40^ which provides a time-dependant offset from the atmospheric IntCal13 curve for the global ocean, and applied a local correction (ΔR) of 64 ± 41 ^14^C yr^41^. The dated specimens were put in relative stratigraphic order and crossmatched with each other following the methods described in ref.^14^ and references therein. Two growth increment chronologies (GIC) were built using ARSTAN^42^ following the methods described in refs.^13,14^. Once crossmatched, the calibrated age range was constrained using the tree ring sequence built into OxCal 4.3^43^. The chronologies developed here only function as a stratigraphic template on which to base the stable isotope geochemical results and are not examined in their own right.

The strength of the chronologies was analysed with the standard dendrochronology and sclerochronology statistic EPS which measures the variance explained by a finite subsample of a population chronology^17^. The EPS is a function of the number of shells contributing to the chronology (sample depth) and the average correlation between shell pairs. A high EPS is usually interpreted as indicating the presence of a strong common environmental signal in the growth increment series of the sampled shell population. An EPS of 0.85 is commonly used as a threshold to indicate that a chronology is reasonably representative of the whole population^17,44^. In this case the EPS was calculated in a 30-year sliding window.

Three of the four shells belonging to the most recent chronology were selected for isotopic analysis at annual resolution. The selection was based on temporal coverage of the individual shells and their taphonomic state, with preference given to the shells that showed the least erosion, the broadest increments and those that provided at least 10 years of temporal overlap between shell pairs. Milling was carried out on the ventral margin on the outer layer of each shell at the School of Ocean Sciences, Bangor University, using a computerised New Wave/Elemental Scientific micromill system fitted with a spherical tungsten carbide dental burr with a diameter of 300 µm at the tip. Rotation speed was limited to 12% (4,450 rpm) to minimise CaCO_3_ polymorph transformation^45^. The entirety of the outer layer in each annual increment was milled between the growth lines to an average depth of 100 µm.

All the powder extracted from a given increment was thoroughly homogenized before an aliquot of the sample was isotopically characterised at the Institute of Geosciences, University of Mainz (Germany) following the methods described in ref.^46^. Isotope data showed a 1σ external reproducibility (accuracy based on 421 NBS-19 samples) better than 0.04‰ for δ^18^O and 0.03‰ for δ^13^C and average internal precision of 0.09‰ for δ^18^O and 0.04‰ for δ^13^C. Both isotope values were reported as per mil deviations relative to the Vienna Pee Dee Belemnite (VPDB) standard. No correction for different acid fractionation factors of shells samples (aragonite) and the reference material (calcite) was applied^47^.

We inspected the results against the average peak intensity given by the mass spectrometer and rejected those that showed abnormally high/low isotope values and those with intensities falling significantly outside the range of the reference materials that were not paired with a higher intensity sample. The δ^18^O and δ^13^C results were weight-averaged into single series before further analysis. The weights were given by 1/σ^2^ where σ represents the internal precision for each sample which is affected by the peak intensity.

δ^18^O and δ^13^C values in intervals where an extended (>10 yr) noticeable change in average occurred were checked for normality using the Shapiro-Wilk W score^48^. The average values in these intervals were compared using a two-tailed t-test and the variance was compared using an F-test. We also assessed the significance of linear trends in these intervals.

We approximated the long-term variability in δ^13^C using a weighted Fourier regression consisting of the largest two coefficients. The weights were given by 1/σ^2^ where σ represents the internal precision for each sample. To emphasise the high frequency variability in δ^13^C, we subtracted the Fourier regression from the weight-averaged results.

We compared our data to the *N. pachyderma* (s) abundance record developed in ref.^6^ from sediment core 28–03 (60.867°N, 3.733°E) which covers the 8.2 ka event in the northern North Sea with an average temporal resolution of 7 years. The age model for this core was updated using the Marine13 calibration curve^40^ with a local ΔR correction of 64 ± 41 ^14^C yr^41^. We arbitrarily used the median of each re-calibrated age range and converted core depth by linearly interpolating the dates. We also compared our results to the sudden relative sea level rise (SSLR) data derived from terrestrial cores from the Cree Estuary, south west Scotland^18^.

## Supplementary information

Supplementary methods

Dataset 1

Photos of 8.2kC shells

Photos of 8.7kC shells
