# Supplementary material for: 8.2 ka event North Sea hydrography determined by bivalve shell stable isotope geochemistry
Source: Sci Rep. 2019 May 1;9:6753. doi: 10.1038/s41598-019-43219-1 (PMC6494846; doi:10.1038/s41598-019-43219-1)
Supplement: Supplementary file 1 — Supplementary methods [file 41598_2019_43219_MOESM1_ESM.docx]

This file contains supplementary methods for:

**8.2 ka event North Sea hydrography determined by bivalve shell stable isotope geochemistry**

Juan Estrella-Martínez^1*^, Philippa L. Ascough^2^, Bernd R. Schöne^3^, James D. Scourse^4^, Paul G. Butler^4^

^1^School of Ocean Sciences, Bangor University, Askew St., Menai Bridge LL59 5AB, United Kingdom *e-mail: juan@es-mar.com

^2^NERC Radiocarbon Facility, Scottish Universities Environmental Research Centre, Rankine Avenue, Scottish Enterprise Technology Park, East Kilbride, G75 0QF

^3^Institute of Geosciences, University of Mainz, J.-J.-Becher-Weg 21, D-55128, Mainz, Germany

^4^College of Life and Environmental Sciences, University of Exeter, Penryn Campus, Penryn TR10 9FE, United Kingdom

**Update to the Grossman and Ku palaeotemperature equation**

The updated equation

|  | $T=150.32 \left[ \pm3.94 \right]-4.35 [\pm0.12] (\delta^{18}O_{aragonite}-\delta^{18}O_{water})$ | (1) |
| --- | --- | --- |

was calculated by employing a reduced major axis regression of the original Grossman and Ku data^1^ after taking into consideration the latest determination of equivalence between the VSMOW and VPDB scales^2^ (δ^18^O_VPDB_ = 0.97001*δ^18^O_VSMOW_ – 29.99 ‰) and does not significantly alter the slope originally calculated by Grossman and Ku^1^. Quantities in square brackets represent the standard error. For a δ^18^O_aragonite_ range of +2.0 ‰ to +4.0 ‰ (VPDB), the difference between the original equation^1^ and Equation 1 is between 0.03 °C and 0.06 °C for the δ^18^O_water_ range found in the modern North Sea^3^ (+0.50 ‰ to -0.40 ‰ VSMOW). This is well within the standard error of estimate of the classic palaeotemperature equation (1.37 °C).

References

1. Grossman, E. L. & Ku, T.-L. Oxygen and carbon isotope fractionation in biogenic aragonite: Temperature effects. *Chem. Geol. Isot. Geosci. Sect.* **59,** 59–74 (1986).

2. Brand, W. A., Coplen, T. B., Vogl, J., Rosner, M. & Prohaska, T. Assessment of international reference materials for isotope-ratio analysis (IUPAC Technical Report). *Pure Appl. Chem.* **86,** 425–467 (2014).

3. Harwood, A. J. P., Dennis, P. F., Marca, A. D., Pilling, G. M. & Millner, R. S. The oxygen isotope composition of water masses within the North Sea. *Estuar. Coast. Shelf Sci.* **78,** 353–359 (2008).
